# Comments on “Mycobiota and Mycotoxins in Traditional Medicinal Seeds from China. *Toxins* 2015, *7*, 3858-3875”— in Attributing Ochratoxin A Biosynthesis Within the Genus *Penicillium* Occurring on Natural Agricultural Produce

**DOI:** 10.3390/toxins8060166

**Published:** 2016-05-31

**Authors:** Peter Mantle, Marina Venturini Copetti, Alan Buddie, Jens Frisvad

**Affiliations:** 1 Centre for Environmental Policy, Imperial College London, London SW7 2AZ, UK; 2 Department of Food Technology and Science, Federal University of Santa Maria, Santa Maria CEP 97105-900, RS, Brazil; marinacopetti@yahoo.com.br; 3 Centre for Agricultural and Biosciences International (CABI), Bakeham Lane, Egham, Surrey TW20 9TY, UK; a.buddie@cabi.org; 4 Department of Systems Biology, Technical University of Denmark, 2800 Lyngby, Denmark; jcf@bio.dtu.dk

**Keywords:** mycotoxicology, single-spore isolates, ochratoxin A, *Penicillium polonicum*, *Penicillium solitum*

## Abstract

The unusual attribution of trace amounts of ochratoxin A in some Chinese food commodities to *Penicillium polonicum* is questioned by European experience in searches for ochratoxinogenic food-spoilage Penicillia, where mistaken attribution is now known to have been due to cryptic *Penicillium verrucosum* contamination. Consequently, selection of single-spore isolates is recommended as pre-requisite for attributing mycotoxin biosynthetic potential to fungi.

In the manuscript in question [1], we find that designation of *Penicillium polonicum* as a biosynthetic source of ochratoxin A (OTA) is open to objective discussion. The topic arises from recognition of *P. polonicum* as a common contaminant of the medicinal plant products, lychee and tangerine seeds, detecting OTA in the seeds, and also quantifying its occurrence in small amounts in rice cultures of the fungus. *P. polonicum* was identified by standard phenotypic characters and ITS sequencing. OTA was recognized by LC/MS. However, the protocol for isolation of pure culture of the fungus is less than rigorous, because monoconidial cultures were not obtained from the original isolate, before mycotoxin investigation was started. 

The reasoning behind this critique arises from experience in analogous studies on ochratoxinogenic fungi in hyperendemic villages in Yugoslavia (Croatia, 1989) and Bulgaria (Vratza, 1991) [2,3]. The purpose of the previous studies was in the context of mycotoxins as putative causal agents of the idiopathic human disease Balkan Endemic Nephropathy occurring in localised agricultural communities. Although OTA was first isolated and characterised from *Aspergillus ochraceus* in South Africa [4], and subsequently recognised as a *Penicillium* metabolite in Europe [5], there have been few *Penicillium* spp. in which OTA biosynthesis has been confirmed [6,7,8]. *P. verrucosum* and *P. nordicum* are both relatively slow-growing moulds with green colour not easily differentiated in laboratory culture from many common Penicillia, particularly the *P. polonicum* which was the most abundant species in the Balkan surveys [2,3]. Notably also, *P. polonicum* is a modern designation arising from rationalising the complexity of the former *P. aurantiogriseum* [9].

Although no ochratoxinogenic Penicillia were found in the Croatian study [2], three fungi from *Zea mays* in two Bulgarian nephropathy households [3] (*P. griseofulvum, P. solitum* and *P. viridicatum*, recognised according to [6] and subsequently illustrated in colour [10]), had been transferred as inoculum to moist shredded wheat for 3 weeks at 18 °C. Sensitive analysis for OTA showed 1.1 and 1.3 µg/kg for *P. griseofulvum* and *P. viridicatum*, respectively, but a much higher yield (1.3 mg/kg) for *P. solitum*. Isolates were then deposited, in 1992, in the CABI IMI Genetic Resources Collection where they were lyophilised and designated as IMI 351308, IMI 351306 and IMI 351304, respectively. 

Archived vials of these isolates have recently been reviewed for homogeneity by systematic spread plate methodology in Brazil, far from any potential European contamination. Contamination was evident in *P. solitum*; contrasting colony diameter on Czapek yeast extract agar at 25 °C for 1 week was 29 mm and 16 mm for the principal component and a contaminant, respectively. Morphologically, the principal conformed to *P. solitum*, while the contaminant had some characteristics of *P. verrucosum* (Figure 1A,B). *P. viridicatum* was also contaminated: whereas the principal generally conformed morphologically to the type (31 mm at 25 °C), its contaminant (Figure 2) unexpectedly also made some growth at 37 °C. Ribosomal DNA internal transcribed spacer regions, incorporating the 5.8S rDNA subunit (hereafter referred to as ‘ITS sequences’), of these isolates were explored, together with partial β-tubulin gene findings, at CABI, UK, where the IMI culture collection is conserved. ITS sequences of principal and contaminant components of *P. solitum* (IMI 351304) both pointed to *P. polonicum*, but it was not possible to resolve them further because of unclean β-tubulin gene sequences. *P. viridicatum* (IMI 351306) ITS sequences were 100% consistent with both *P. viridicatum* and *P. verrucosum*, and 99.8% similar to *P. polonicum*, but β-tubulin sequence data matched *P. viridicatum* at 100 %. However, *P. solitum*, *P. polonicum*, *P. viridicatum* and *P. verrucosum* are all in *Penicillium* section *Viridicata*. We conclude that the isolated fungi [3] designated as Penicillia and named according to the recognised diagnostic criteria at the time, and to which OTA biosynthesis was attributed, were mixtures. Consequently, the importance of making single-spore isolates, however additionally laborious, is emphasised as essential rigour for attributing mycotoxinogenicity. Recommendation concerning necessary rigour in attributing OTA biosynthesis within Penicillia can extend also to [11]. 

Independently, IMI 351304 had also been deposited in Denmark. Subsequently, critical review of its homogeneity preferred the identity as *P. polonicum* (IBT 14320) but also found *P. verrucosum* (IBT 14248) as a contaminant, consistent with the conclusion of the above studies on IMI 351304 in Brazil and the UK. The strains of *P. griseofulvum* (IMI 351308 = IBT 14319) and *P. viridicatum* (IMI 351306 = IBT 14245) were also purified and their identity confirmed, but OTA could not be detected in any of those two isolates. Thus in the genus *Penicillium* it is still the case that only *P. verrucosum* and *P. nordicum* have been convincingly shown to be able to produce ochratoxin A. Screening for OTA in a large number of isolates of *P. polonicum*, *P. griseofulvum* and *P. viridicatum* has never revealed any trace of OTA [8].

We do not doubt the occasional occurrence of OTA in lychee and tangerine seed, or in liquorice [12], in China [1], or also in agricultural commodities worldwide. However, from our experience above, we find attribution of OTA biosynthesis to *P. polonicum* unsafe without the rigour of testing single-spore isolates. The analogous situation concerning OTA and Balkan endemic nephropathy [2,3,13] is equally unsatisfactory in being unsure what Penicillia might contribute to the traces of OTA in foodstuffs found, not exclusively, in hyperendemic villages. *P. verrucosum* was found occasionally but did not produce OTA in the laboratory. We are not aware of *P. nordicum* being isolated from nephropathy households in continental Balkan regions. However, a notable study defining the source of this ochratoxinogenic contaminant on dried salted meat products manufactured in Western Slovenia [14] proved that local maritime sea-salt provided the inoculum. The fungus has also been isolated from meat products across several Mediterranean latitudes. *P. polonicum* was the most abundant mould contaminant in previous Balkan nephropathy studies [2,3]; although never found to produce OTA, it did commonly produce a novel nephrotoxin, expressed notably in the rat but not the primate [15,16,17,18]. It would be interesting if this species in China has similar toxigenic potential. Nevertheless, it would be unfortunate to attribute to one of the most ubiquitous food-spoilage moulds the ability to produce OTA, a potent renal carcinogen for the male rat, if this is actually not correct. 

## Figures and Tables

**Figure 1:**
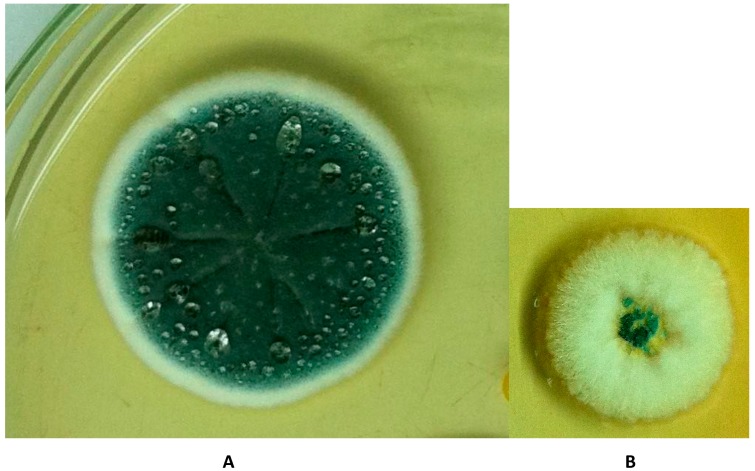
Colonies of principal (**A**) and contaminant (**B**) components of *P. solitum* (IMI 351304) grown on Czapek Yeast Agar at 25 °C for 7 days, displayed in relative size.

**Figure 2:**
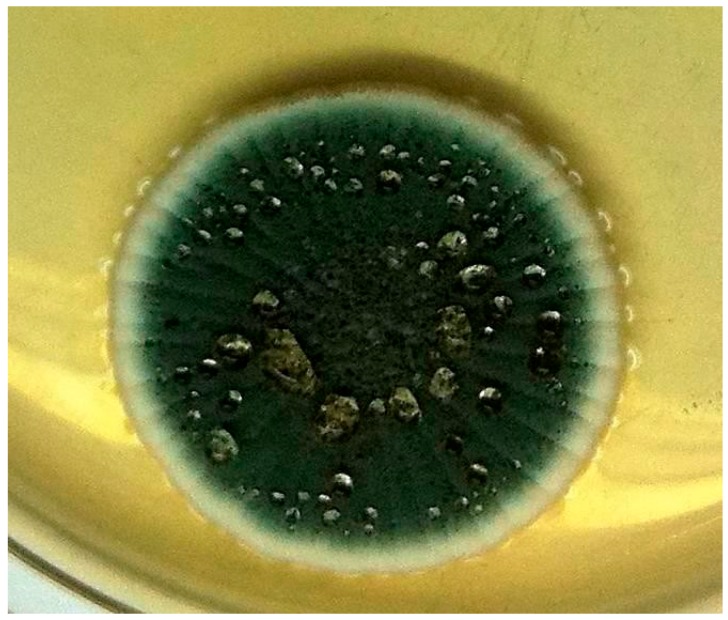
Colony of contaminant of *P. viridicatum* (IMI 351306), size relative to Figure 1.
